# Response of maize yield and nitrogen recovery efficiency to nitrogen fertilizer application in field with various soil fertility

**DOI:** 10.3389/fpls.2024.1349180

**Published:** 2024-02-27

**Authors:** Hongqin Zou, Dejin Li, Keyu Ren, Lisheng Liu, Wenju Zhang, Yinghua Duan, Changai Lu

**Affiliations:** ^1^ State Key Laboratory of Efficient Utilization of Arid and Semi-arid Arable Land in Northern China/Institute of Agricultural Resources and Regional Planning, Chinese Academy of Agricultural Sciences, Beijing, China; ^2^ Red Soil Experiment Station of Chinese Academy of Agricultural Science in Hengyang/National Observation and Research of Farmland Ecosystem in Qiyang, Qiyang, China

**Keywords:** red soil, nitrogen fertilizer application rate, nitrogen use efficiency, soil microbial biomass carbon, soil microbial biomass nitrogen

## Abstract

Appropriate nitrogen (N) management system is essential for effective crop productivity and minimizing agricultural pollution. However, the underlying mechanistic understanding of how N fertilizer regulates crop yield via soil properties in soils with different fertilities remains unresolved. Here, we used a field experiment that spanned 3 cropping seasons to evaluate the grain yield (GY), aboveground biomass and N recovery efficiency (NRE) after treatment with five N fertilizer application rates (N0, N75, N112, N150, and N187) in soils with three levels of fertility. Our results indicated that the highest GY across low, moderate, and high fertility soils were 1.5 t hm^-2^ (N150), 4.9 t hm^-2^ (N187), and 5.4 t hm^-2^ (N112), respectively. The highest aboveground biomass and NRE were observed at N150 for all three levels of soil fertility, while only the N uptake by aboveground biomass of low and high fertility soils decreased at N187, confirming that excessive N fertilization results in a further decline in crop N uptake. The relationship between GY, NRE and N fertilizer application rates fit the unary quadratic polynomial model. To achieve a balance between grain production and environmental benefits in N fertilizer, appropriate N fertilizer rates were determined to be 97.5 kg hm^-2^, 140 kg hm^-2^ and 131 kg hm^-2^ for low, moderate and high fertility soils, respectively. Structural equation modeling suggested that GY was significant correlated with soil microbial biomass carbon (SMBC) and N directly in low fertility field, with SMBC directly in moderate fertility field, and via SOC and NO_3_^–^N in high fertility field. Therefore, a soil-based management strategy for N fertilizers could enhance food security while reducing agricultural N fertilizer inputs to mitigate environmental impacts.

## 1 Introduction

Nitrogen (N) fertilizer has been widely used in agriculture and is essential for increasing agricultural productivity on a global scale (Lassaletta et al., 2014). Sufficient N fertilization is the basis for increasing crop yield and population growth. However, the excessive application of N fertilizer to farmland to increase crop production over an extended period of time has caused a cascade of problems, including not only a decrease in grain yield but also an increase in environmental and economic burden in agricultural ecosystems (Fan et al., 2018). Therefore, finding the appropriate N fertilizer application rate is crucial to ensure high crop yields while minimizing environmental N loss (Zhang et al., 2018).

Under the premise of ensuring high crop yield, researchers have developed estimation models based on field N fertilizer efficiency experiments to predict the appropriate N fertilizer application rate for crops (Yang et al., 2019). Wang et al. (2014) suggested an appropriate N fertilizer application rate was 289 kg hm^-2^ for the highest maize yield, regardless of soil fertility. While Zou et al. (2022) indicated that the appropriate N fertilizer application rates for maize were 170–235 kg hm^−2^, depending on the soil fertility level, to achieve maximum yields and N use efficiency. As soil fertility determines the amount of N available to crops, it also controls the soil-plant-microbial interactions via the balance/imbalance among soil chemistry, physics, and soil biology (Uphoff et al., 2013). Therefore, soil fertility should be considered in determining appropriate N fertilizer application rates and N fertilizer management in agricultural fields.

Several studies have indicated that improvement of soil fertility was conductive to reducing crop dependence on N fertilizer, resulting in a reduction in the amount of N fertilizer needed to maximize crop yields (Yang et al., 2014; Cai et al., 2019). However, there were differences in the conclusions regarding soil fertility’s effect on N recovery efficiency (NRE). Liao et al. (2010) reported that both N fertilizer contribution rate and NRE of crops were low in soils with high fertility. On the contrary, a higher NRE was observed in high fertility soil compared to that in low fertility soil in Beatriz et al. (2017). The main explanation for these differences is that appropriate N fertilizer application rate should depend on the soil fertility, and this rate should differ for different levels of soil fertility. Therefore, to meet the demands for food security and environment protection, it is necessary to establish the quantitative relationship between crop yield and NRE with N fertilizer application rates for different levels of soil fertility.

The factors which limit grain production are varied in low, moderate, and high fertility soils. Lu et al. (2022) indicated that the main limiting factor in low fertility soil was carbon storage, which restricts the turnover of soil nutrients (Malik et al., 2020). Soil microbial biomass N (SMBN) was found to be an important factor in high fertility soil (Liu et al., 2008) because its formation could not only retain organic N but also affect the availability of soil N by regulating soil nitrification potential (Wang et al., 2019b). However, knowledge and understanding of the relationship between grain yield (GY) and N fertilizer application rate is lacking, and the key factors for the response of grain production to N fertilizer application is not well understood in low, moderate, and high fertility soils.

Our study was designed to establish a relationship between GY, NRE and N fertilizer application rates, and to exploit the most important soil properties in low, moderate, and high fertility soils. Using a 3-crop season field experiment, the GY of maize, N uptake by aboveground biomass, and NRE were examined under five N fertilizer application rates in soils with three different levels of soil fertility. The specific objectives of this work were to: (1) Determine the response of GY and NRE to different amounts of N fertilizer application in low, moderate, and high fertility soils; (2) Establish the relationship between the GY of maize, NRE and N fertilizer application rates in soils with different levels of fertility; (3) Better understand how N fertilizer application rate alters crop yield via soil properties in three levels of soil fertility.

## 2 Materials and methods

### 2.1 Site description

The experiment was conducted at the Red Soil Experimental Station in Qiyang, Hunan Province, China (26° 02′ N, 110° 35′ E) from 2019. This region is characterized by a typical subtropical monsoon climate with mean annual air temperature of 18.0°C, mean annual precipitation of 1 250 mm, and mean annual evaporation of 1 470 mm (data from China meteorological sharing service system, http://cdc.cma.gov.cn/). The soil is originated from quaternary red clay and is categorized as Eutric Cambisol according to FAO classification with a silty clay texture. The initial SOC of 0–20 cm topsoil before experimental treatment were 7.67–28.30 g kg^-1^, and pH, Alkaline N, Olsen-P, and available K varied at 5.35–6.74, 37.53–144.4 mg kg^-1^, 0.20–23.55 mg kg^-1^, and 18.89–173.9 mg kg^-1^, respectively (**Table 1**).

**Table 1:** Initial properties of soil with low, moderate and high fertility (before the experiment in 2019).

Soil fertility	Organic carbon/(g·kg^-1^)	pH	Total N /(g·kg^-1^)	Alkaline N/(mg·kg^-1^)	Total P /(g·kg^-1^)	Olsen-P/(mg·kg^-1^)	Total K/(g·kg^-1^)	Available K/(mg·kg^-1^)
Low	7.67 ± 0.40c	5.35 ± 0.02c	0.64 ± 0.06c	37.53 ± 2.32c	0.31 ± 0.01c	0.20 ± 0.03c	10.12 ± 0.11c	18.89 ± 1.90c
Moderate	11.69 ± 0.22b	6.04 ± 0.12b	0.87 ± 0.02b	62.25 ± 2.84b	0.54 ± 0.02b	11.76 ± 0.41b	12.48 ± 0.21a	173.9 ± 11.78a
High	28.30 ± 0.21a	6.74 ± 0.03a	1.72 ± 0.21a	144.4 ± 3.11a	0.84 ± 0.01a	23.55 ± 0.33a	10.8 ± 0.10b	156.3 ± 2.05b

Values are the mean ± SD (standard deviation). Low, Moderate, and High represent low fertility soil, moderate fertility soil, and high fertility soil, respectively. Different lowercase letters indicate significant difference at 5% level.

### 2.2 Experimental design and treatment

The crop system was winter wheat (*Triticum Aestivium* L.) and summer maize (*Zea mays* L.) rotation. In 2019, the experiment consisted of a fertility gradient with three soil organic carbon (SOC) contents: low (7.67 g kg^-1^), moderate (11.69 g kg^-1^), and high (28.30 g kg^-1^) according to Bulletin of the Ministry of Agriculture and Rural Affairs (2020a), with five N fertilizer application rates. Therefore, there were 15 treatments, each with three replicates, in a completely randomized block design. The N fertilizer application rates were 0 kg hm^-2^ (N0), 75 kg hm^-2^ (N75), 112.5 kg hm^-2^ (N112), 150 kg hm^-2^ (N150), and 187.5 kg hm^-2^ (N187) for maize, and 0 kg hm^-2^, 37.5 kg hm^-2^, 56.25 kg hm^-2^, 75 kg hm^-2^, and 93.75 kg hm^-2^ for wheat. The application amounts of phosphate fertilizer (P_2_O_5_) and potassium fertilizer (K_2_O) were 75 kg hm^-2^ for maize and 38 kg hm^-2^ for wheat. N fertilizer was urea (N content 46%), phosphate fertilizer was superphosphate (P_2_O_5_ content 12.0%), potassium fertilizer was potassium chloride (K_2_O content 60%), all of which were used as a single application base fertilizers before sowing. The treatments were initially randomized at each study site and remained consistent on the same plot in subsequent cropping seasons. Winter wheat was sown in early November and harvested in mid-May, summer maize was sown in late May to early June and harvested in mid-August. None of the stover harvested from the plots was returned to the field. Each plot was 6 m^2^ (2 m by 3 m), and the plots were separated by 100-cm-deep cement baffle plates.

### 2.3 Soil and plant sampling and analysis

After maize and wheat crop maturity in August 2020 and May 2021 (the third and the fourth cropping season in this experiment), the plants were manually harvested using sickles and all biomass was removed from all the treatment plots. The whole plot was used to determine the Grain yields (GY). Additionally, five samples of stover and grain were taken from each treatment plot and analyzed separately. The N content of crops were determined after drying and threshing the plants. The aboveground biomass was calculated as the sum of stover and grain biomass, while total N uptake was determined by adding the N uptake by both stover and grain. Belowground roots were excluded from N uptake and N recovery efficiency (NRE) calculations due to the sampling challenges involved.

Soil was also sampled at this time. Five soil cores were randomly sampled from each treatment (0–20 cm) using a 2.5-cm diameter auger and mixed to produce a composite soil sample. The soil samples were transported to the laboratory on ice immediately after collection. Fresh soil was sieved through a 2-mm sieve and any plant litter and stones were manually removed. The soil samples were divided into two portions, with one half designated for the determination of soil nutrient contents, and the other half was used for determination of soil microbial biomass carbon (SMBC) and SMBN.

Soil moisture content was determined gravimetrically after oven drying fresh soil in aluminum dishes for 8 hours at 105 °C. Soil pH was measured at a ratio of dry soil to water ratio of 1:2.5 (w:v) by a pH electrode. Soil organic carbon (SOC) was determined by vitriol acid-potassium dichromate oxidation (Walkley, 1935). Soil total N (TN) was determined by the Kjeldahl digestion-distillation method (Black, 1965). Soil total phosphorus (TP) was digested with HClO_4_–H_2_SO_4_ and determined using the molybdenum blue colorimetric method (Murphy and Riley, 1962). Soil total potassium and available potassium were determined by flame photometer (Lu, 2000). Soil alkaline N (AN) was determined by alkaline solution diffusion (Lu, 2000), and soil Olsen-P was performed by Olsen method (Olsen, 1954). Soil mineral N (ammonium and nitrate) contents were quantified by colorimetric methods as described by Hood-Nowotny et al. (2010).

Soil microbial biomass C (SMBC), and SMBN were determined by the chloroform fumigation extraction method described by Wu et al. (1990). Briefly, 30 g of each soil sample was adjusted to 60% of field water holding capacity (WHC) through the addition of sterile deionized water, and pre-incubated at 25 °C in the dark for one week. Then, 12.5 g of each sample was fumigated with ethanol-free chloroform for 24 h in the dark at 25 °C, these samples were paired with 12.5 g soil samples that were not fumigated but incubated for 24 h under the same conditions. Total dissolved organic C and total dissolved N were extracted in 50 mL 0.5 M K_2_SO_4_ from fumigated and unfumigated soil samples and analyzed with a TOC/TN analyzer (muti C/N 3100, Germany). The conversion coefficients used to calculate the SMBC, and SMBN were 0.45 and 0.54, respectively. Post harvest soil properties are shown in **Supplementary Table A1**.

### 2.4 Data calculation and statistical analysis

Yield and N efficiency were key parameters to evaluate the efficacy. To determine the proportion of applied N fertilizer assimilated in the harvested parts, N recovery efficiency (NRE) was calculated using equation (1) (Salvagiotti et al., 2009).


$$NRE=\frac{harvest\;yield\;N\;uptake\;in\;fertilized\;plots-harvest\;yield\;N\;uptake\;in\;unfertilized\;plots}{the\;amount\;of\;fertilizer\;N\;applied}$$

Two-way analysis of variance (ANOVA) was performed by SPSS 20.0 (SPSS Inc., Chicago, IL, USA) for windows to analyze the effects of N fertilization in three fertility soils followed by Ducan test, and *p*< 0.05 was considered as the threshold value for significance. Nonlinear regression analysis was used to fit the relationship between GY, NRE and N fertilizer application rates in the three levels of soil fertility. The data for GY and soil properties were fitted with linear regression: y = ax +b, where a and b were two parameters.

Structural equation model (SEM) was further performed by using the Amos 21.0 software package (Amos Development Corporation, Chicago, IL, USA) to examine how GY was affected by N fertilizer application rates and soil properties through hypothetical pathways. This approach can partition isolate the direct and indirect effects that one variable may have on another (Hershberger, 2001; Grace, 2006). In this study, we analyzed the linear regression relationships between GY and soil properties, and found that soil pH, SOC, nitrate (NO_3_^-^), SMBC, and SMBN contents were significantly correlated with GY. Therefore, the above soil properties and GY were incorporated into the SEM. Using a recursive model with a sample size of 15, the best-fit model was obtained through maximum likelihood. The model fit was assessed and determined by a chi-square test (χ^2^), low χ^2^/df (< 3), *P* values (0.05< *P*< 1), high increment-of-fit index (IFI > 0.9), and low root mean square errors of approximation (*RMSEA*). Iterative improvements to the model fit were made by adding or removing relationships between observed variables based on modification indices from prior models. All graphs were prepared by using Origin 2021 software (Origin Lab Corporation, Northampton, MA, USA).

## 3 Results

### 3.1 Increased yield and aboveground biomass through nitrogen fertilizer application and soil fertility

**Figure 1:**
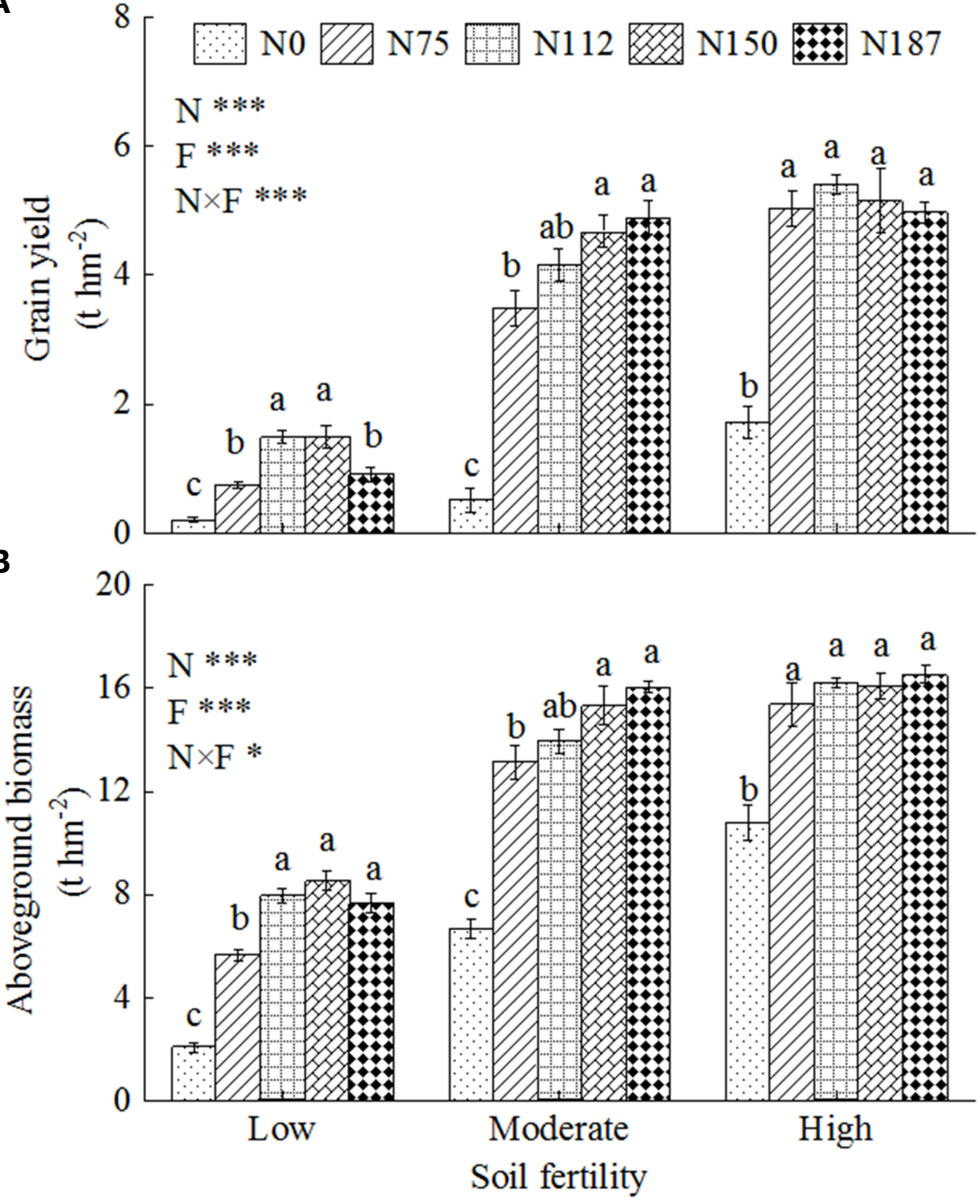
Grain yield of maize **(A)** and aboveground biomass of maize **(B)** for three levels of soil fertility with N fertilizer application gradients. N0, N75, N112, N150, and N187 indicate nitrogen fertilization rates of 0, 75, 112.5, 150, and 187.5 kg N hm^-2^, respectively. Low, Moderate, and High represent low fertility soil, moderate fertility soil, and high fertility soil,. Different lowercase letters indicate significant difference at 5% level. F, N and F×N represent soil fertility, nitrogen fertilizer application and interaction effect of soil fertility and nitrogen fertilizer, respectively. * and *** represent *P*< 0.05 and *P*< 0.001.

The effects of different N fertilizer application rates on maize growth, grain yield (GY) and aboveground biomass were examined (**Figure 1**). The response of GY to N fertilizer application rates was different for the three levels of soil fertility (**Figure 1A**). The highest GY (1.5 t hm^-2^) was observed for N112 and N150 in low fertility soil, and then decreased for the 187 kg hm^-2^ N fertilizer rate. In moderate fertility soil, GY increased significantly with higher N fertilizer application rates, reaching a maximum at 4.7 t hm^-2^ and 4.9 t hm^-2^ for N150 and N187 treatments, respectively. In high fertility soils, the GYs (5.0–5.4 t hm^-2^) for treatments with N fertilizer were significantly higher than the treatment without N fertilizer (N0), while there was no significant difference among N75–N187 for the fertility soil.

The aboveground biomass showed a similar trend with GY (**Figure 1B**). However, the relative gap between soils was smaller than for GY. For example, the highest biomass for moderate and high fertility soil was about 2 times of that of low fertility soil, while it was more than 3 times for GY, indicating improved soil fertility primarily benefited grain production rather than stover formation. The highest aboveground biomasses were obtained at N112, N150, and N75 in low, moderate and high fertility soils, respectively. The maximum maize biomass in low, moderate and high fertility soils were 7.9 t hm^-2^, 15.4 t hm^-2^, and 15.4 t hm^-2^, respectively, which were 2.8, 1.3, and 0.4 times higher than those of the N0 treatment in the same level of soil fertility, respectively.

### 3.2 Nitrogen uptake by aboveground biomass for the three levels of soil fertility

**Figure 2:**
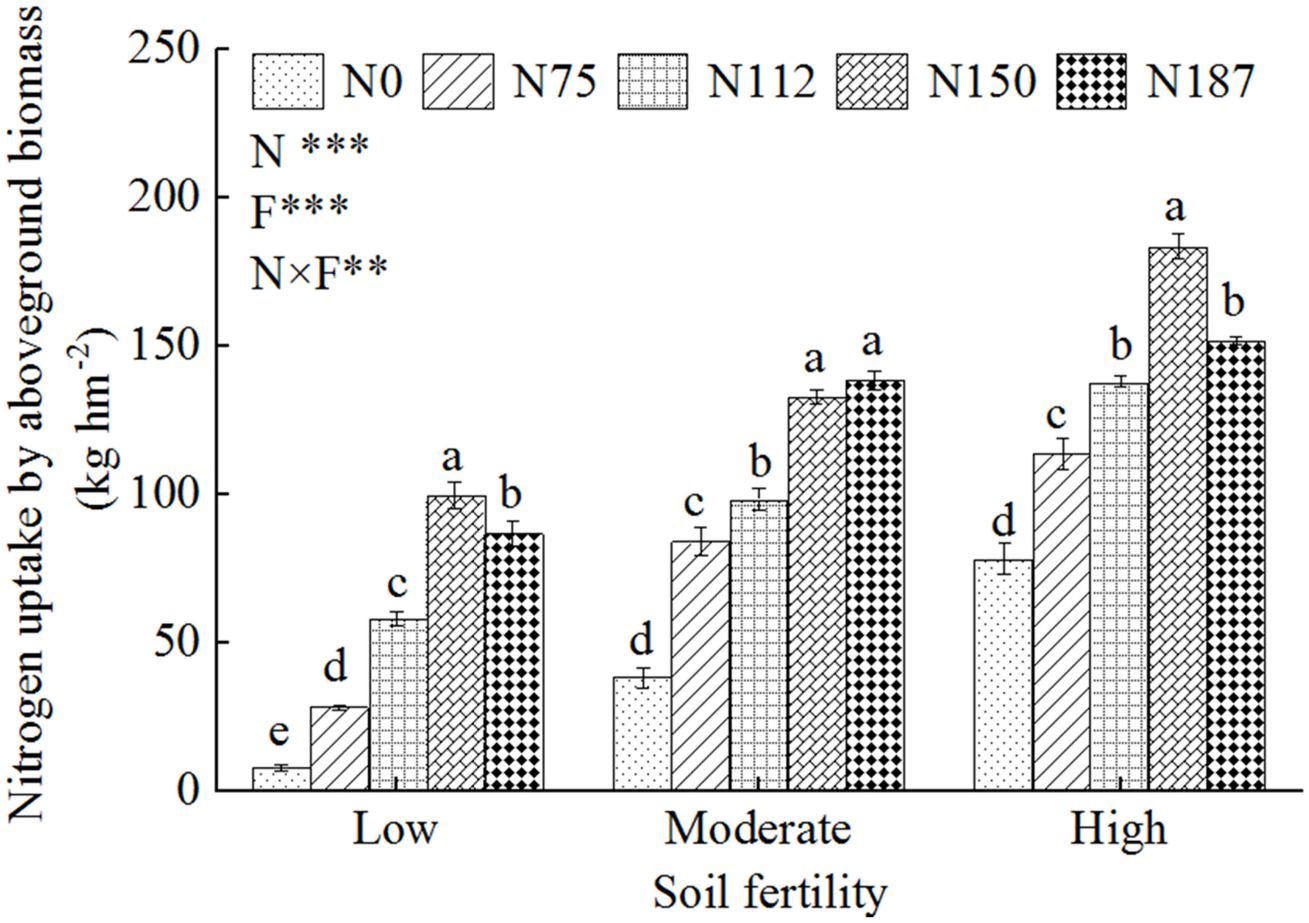
Nitrogen uptake by aboveground biomass for three levels of soil fertility with N fertilizer application gradients. N0, N75, N112, N150, and N187 indicate nitrogen fertilization 0, 75, 112.5, 150, and 187.5 kg N hm^-2^, respectively. Low, Moderate, and High represent low fertility soil, moderate fertility soil, and high fertility soil, respectively. Different lowercase letters indicate significant difference at 5% level. F, N and F×N represent soil fertility, nitrogen fertilizer application and interaction effect of soil fertility and nitrogen fertilizer, respectively. ** and *** represent *P*< 0.01 and *P*< 0.001.

Under N fertilizer application rates of 0–150 kg hm^-2^, the N uptake by aboveground biomass increased significantly (*P*< 0.05) with increasing N fertilizer rate for all three levels of soil fertility (**Figure 2**). The highest N uptake were 99.5, 132.6, and 183.4 kg hm^-2^ for N150 in low, moderate, and high fertility soils, respectively. It should be noted that the highest N uptake for N150 in low fertility soil was similar with that for N112 in moderate fertility soil, and the highest N uptake for N150 in moderate fertility soil was also similar with that for N112 in high fertility soil, which indicated that achieving soil fertility improvement goals would allow reduction of the N fertilizer application rate. When the N fertilizer application rate was increased from 150 kg hm^-2^ to 187 kg hm^-2^, the N uptake of aboveground biomass was significantly reduced in low and high fertility soils, while no significantly change was observed in moderate fertility soil. These results confirmed that overfertilizing with N in excess of the crop requirement leads to a further reduction in N uptake by the crop.

### 3.3 Nitrogen recovery efficiencies at different levels of soil fertility

**Figure 3:**
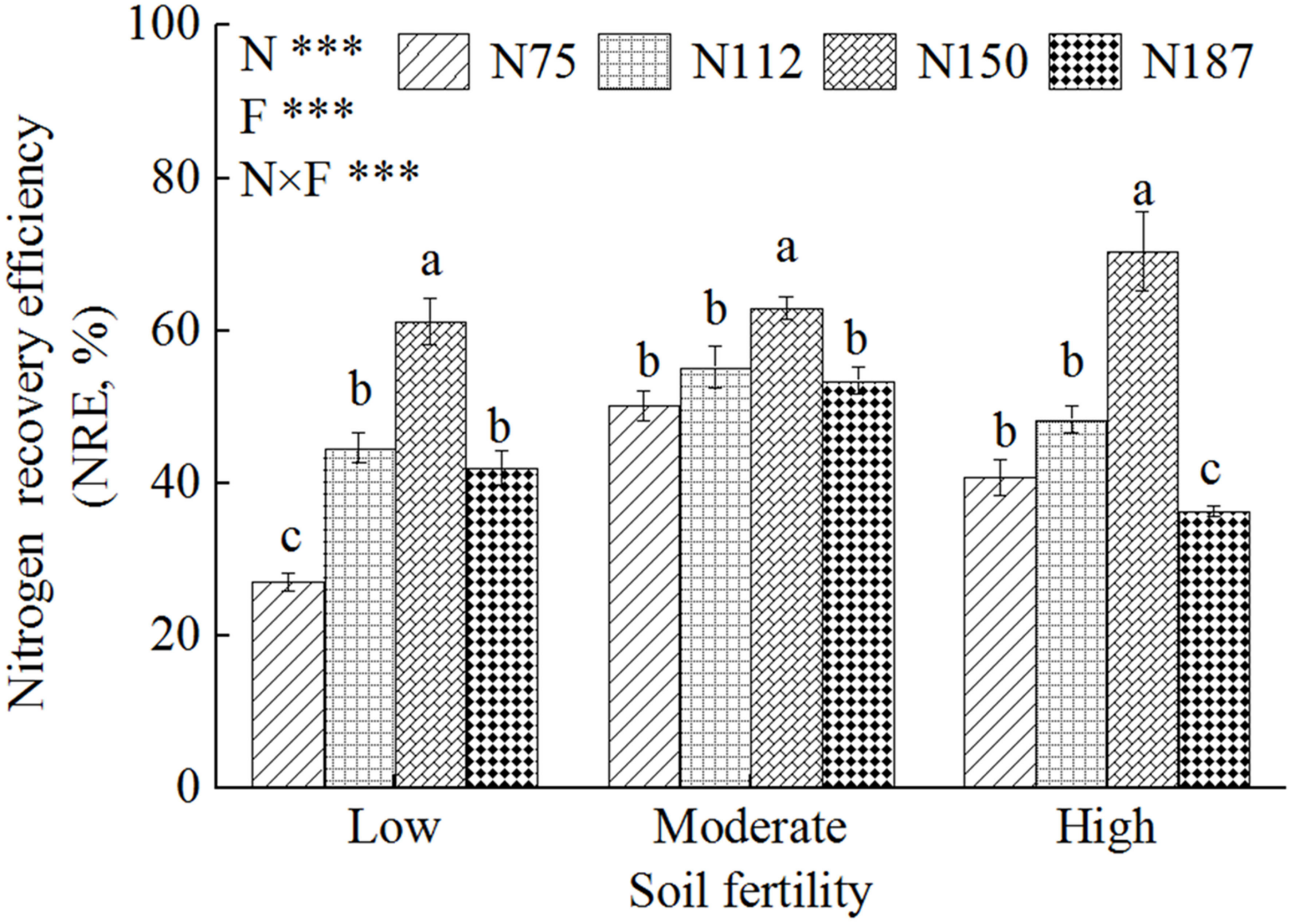
Nitrogen recovery efficiency of maize for three levels of fertility soils with N fertilizer application gradients. N0, N75, N112, N150, and N187 indicate nitrogen fertilization 0, 75, 112.5, 150, and 187.5 kg N hm^-2^, respectively. Low, Moderate, and High represent low fertility soil, moderate fertility soil, and high fertility soil, respectively. Different lowercase letters indicate significant difference at 5% level. F, N and F×N represent soil fertility, nitrogen fertilizer application and interaction effect of soil fertility and nitrogen fertilizer, respectively. *** represent *P*< 0.001.

To evaluate the economic and environmental benefits of N fertilizers, N recovery efficiency (NRE) was analyzed (**Figure 3**). The highest NREs were obtained with the N150 treatment, which were 61.2%, 62.9%, and 70.4% in low, moderate, and high fertility soils, respectively. In low fertility soil, the NRE of the N112 and N187 treatments were similar, and significantly higher (55%–64%) than the N75 treatment. There were no significant differences in NRE among N75, N112, and N187 for moderate fertility soil. In high fertility soil, the NRE for N75 and N112 were significantly lower than that for N150 (41–48%), but higher than that for N187 (36.4%).

### 3.4 Relationships between nitrogen fertilizer application rate and grain yield and nitrogen recovery efficiency

**Figure 4:**
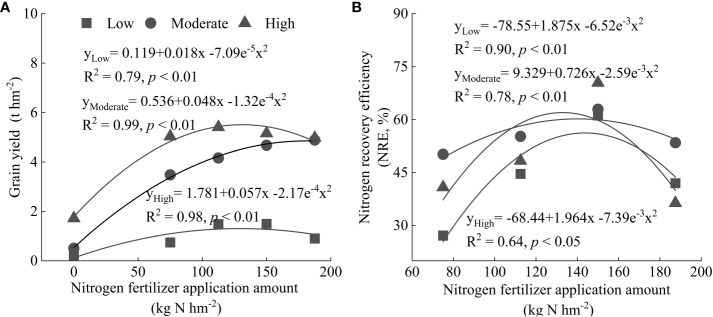
Relationship of nitrogen fertilizer application rate with grain yield **(A)** and nitrogen recovery efficiency **(B)**. Low, Moderate, and High represent low fertility soil, moderate fertility soil, and high fertility soil, respectively.

In the multiple regression analysis of grain yield (GY), N recovery efficiency (NRE) and N fertilizer application rate, unary quadratic polynomial models for GY and NRE of maize were selected because of the higher determination coefficients (R^2^) (**Figure 4**). These quantitative relationships showed that an increase in the N fertilizer application rate could improve GY and NRE within a specific range, but GY and NRE were reduced when the N fertilizer application rate was higher than a specific threshold value. The GYs of low, moderate, and high fertility soils reached their highest points, 1.26 t hm^-2^, 4.90 t hm^-2^, and 5.52 t hm^-2^, when the N fertilizer application rates were 126.9 kg hm^-2^, 181.8 kg hm^-2^, and 131.3 kg hm^-2^, respectively (**Figure 4A**).

Next, the GY, N fertilizer application rate, and corresponding NRE in the three scenarios of maximum GY, 95% of maximum GY (better economic and environmental benefits of N fertilizers), and maximum NRE were analyzed for the low, moderate, and high fertility soils (**Table 2**). In high fertility soil, the maximum GY (5.52 t hm^-2^) and NRE (62%) were achieved simultaneously with 132 kg hm^-2^ N fertilizer. The N fertilizer application rate to achieve the maximum GY was higher in moderate fertility soil (181.8 kg hm^-2^) than that of low and high fertility soils. However, the 95% of maximum GY and maximum NRE (60%) were achieved simultaneously with 140 kg hm^-2^ N fertilizer. For low fertility soil, the GY was similar in the three scenarios but the N fertilizer application rate to achieve maximum GY and NRE was much higher than for 95% of maximum GY, suggesting a limitation of yield improvement by N fertilizer in soil with low fertility. These data indicated that management strategies for N fertilizer should be tailored according to the soil condition in order to enhance the GY and NRE of maize simultaneously.

**Table 2:** Grain yield, nitrogen fertilizer application rate, and corresponding nitrogen recovery efficiency in three scenarios of maximum grain yield, 95% of maximum grain yield, and maximum nitrogen recovery efficiency.

Soil fertility	Maximum grain yield			95% of maximum grain yield			Maximum nitrogen recovery efficiency		
	GY_max_	NFAR-GY_max_	NRE-GY_max_	95% GY_max_	NFAR-95% GY_max_	NRE-95% GY_max_	GY-NRE_max_	NFAR-NRE_max_	NRE_max_
	(t hm^-2^)	(kg hm^-2^)	(%)	(t hm^-2^)	(kg hm^-2^)	(%)	(t hm^-2^)	(kg hm^-2^)	(%)
Low	1.26	126.9	54.4	1.20	97.5	42.5	1.24	143.8	56.2
Moderate	4.90	181.8	55.7	4.66	139.2	60.2	4.67	140.2	60.2
High	5.52	131.3	62.0	5.24	95.2	51.5	5.52	132.9	62.1

GY_max_, NFAR-GY_max_, NRE-GY_max_, 95% GY_max_, NFAR-95% GY_max_, NRE-95% GY_max_, GY-NRE_max_, NFAR-NRE_max_ and NRE_max_ represent the maximum grain yield of maize, the nitrogen fertilizer application rate that grain yield reached the maximum, the nitrogen recovery efficiency that grain yield reached the maximum, 95% of maximum grain yield, the nitrogen fertilizer application rate that grain yield reached the 95% of maximum grain yield, the nitrogen recovery efficiency that grain yield reached 95% of maximum, the grain yield that nitrogen recovery efficiency reached the maximum, the nitrogen fertilizer application rate that nitrogen recovery efficiency reached the maximum and the maximum nitrogen recovery efficiency of aboveground biomass, respectively. Low, Moderate, and High represent low fertility soil, moderate fertility soil, and high fertility soil, respectively.

### 3.5 Linear relationship between grain yield and soil properties

**Figure 5:**
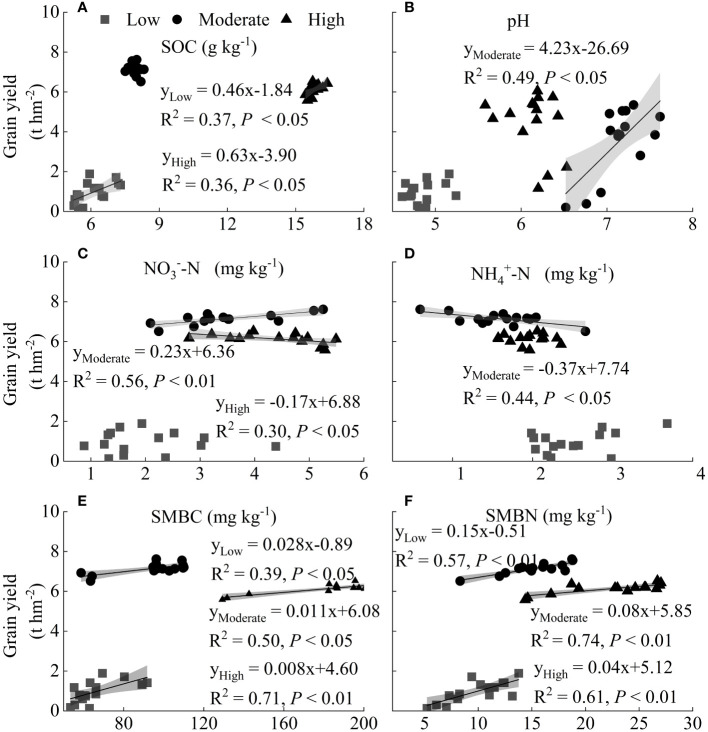
Linear relationship between maize grain yield and SOC **(A)**, pH **(B)**, nitrate **(C)**, ammonium **(D)**, SMBC **(E)** and SMBN **(F)**. Grey shading indicates 95% confidence interval. Low, Moderate, and High represent low fertility soil, moderate fertility soil, and high fertility soil, respectively. SOC, SMBC, and SMBN represent soil organic carbon, soil microbial biomass carbon, and soil microbial biomass nitrogen, respectively.

Linear coefficient analysis was used to investigate the relationship between soil properties and grain yield (GY) under different application rates of N fertilizer (**Figure 5**). Both SMBC and SMBN were significantly positively related with GY (**Figures 5E, F**). The GY was increased by 28, 11, and 8 kg hm^-2^, when SMBC increased by 1 mg kg^-1^, in low, moderate, and high fertility soils, respectively. For every 1 mg kg^-1^ increase in SMBN, the increment of GY was similarly the highest in low fertility soil (150 kg hm^-2^), followed by 80 kg hm^-2^ in moderate, and the lowest in high fertility soil (40 kg hm^-2^). There was a positive linear relationship between soil organic carbon content (SOC) and GY both in low and high fertility soils (**Figure 5A**). Soil pH value was positively related with GY only in the moderate fertility soil (**Figure 5B**). Soil nitrate (NO_3_^–^N) content was positively correlated with GY in moderate fertility soil, but negatively in high fertility soil, and ammonium (NH_4_^+^-N) was negatively related with GY in moderate fertility soil (**Figures 5C, D**).

### 3.6 The influence of nitrogen fertilization on grain yield via soil properties

**Figure 6:**
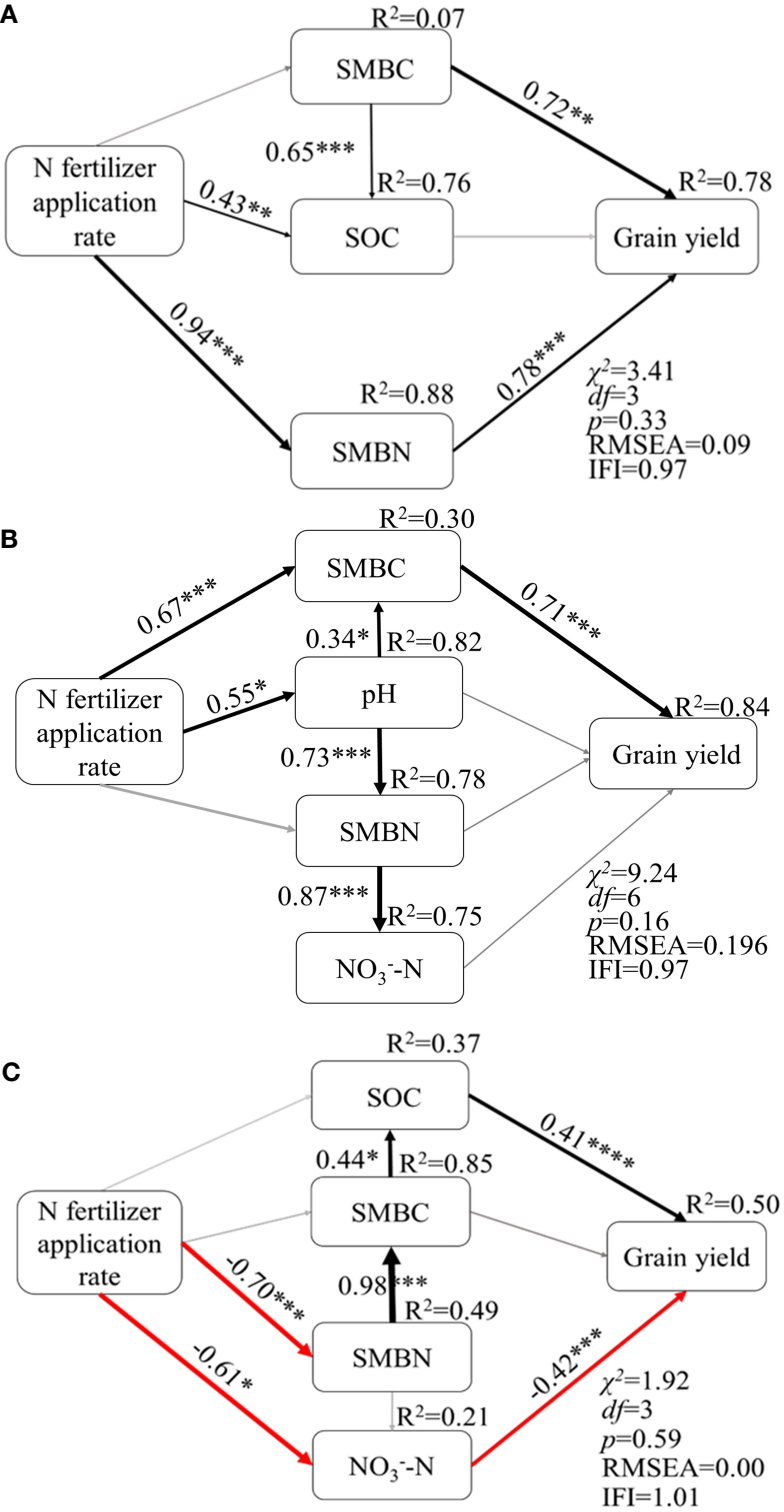
Path analysis of grain yield and soil properties in low **(A)**, moderate **(B)** and high **(C)** fertility soils. * indicates significant effect (*P*< 0.05); ** indicates extremely significant effect (*P*< 0.01); *** indicates extremely significant effect (*P*< 0.001); **** indicates extremely significant effect (*P*< 0.0001). The black and red solid arrows indicate positive and negative effects, respectively, and the grey solid arrows indicate non-significant effects. The numbers adjacent to the arrows are standardized path coefficients, which are indicative of the effect size of the relationship. The proportion of explained variance (R^2^) appears above every response variable in the model. The goodness-of-fit statistics for the model are shown in the lower right corner, *RMSEA*: Root mean square error of approximation.

Given the above findings that soil properties strongly influenced grain yield (GY) and the main factors were different among the three levels of soil fertility, we carried out structural equation modeling (SEM) to link N fertilizer application rate and soil properties with GY of maize (**Figure 6**). The soil properties which correlated with GY in the above section were included in the model.

The established SEM explained 78% of the total variation in GY in low fertility soil (*χ^2 = ^* 3.41; Fisher’s C statistic *P* = 0.33, IFI = 0.97, *RMSEA* = 0.09, **Figure 6A**). N fertilizer application rate had an indirect positive correlation with GY through its impact on SMBN. The high path coefficients for SMBN and SMBC (path coefficients = 0.78 and 0.72, respectively) with GY indicated that soil microbial biomass and activity might be a limitation on the GY of maize in low fertility soil.

In moderate fertility soil, the established SEM explained 84% of the total variation in GY of maize (χ^2 = ^9.24; Fisher’s C statistic *P* = 0.16, IFI = 0.97, *RMSEA* = 0.196, **Figure 6B**). SMBC was directly correlated with GY (path coefficient = 0.71). N fertilizer application rate indirectly affected GY through impacts on pH and SMBC contents (path coefficients = 0.55 and 0.67, respectively). The pH value directly correlated with both SMBC and SMBN.

The established SEM explained 50% of the total variation in GY in high fertility soil (χ^2 = ^1.92; Fisher’s C statistic *P* = 0.59, IFI = 1.01, *RMSEA* = 0.00, **Figure 6C**). SOC was positive correlated with GY, while NO_3_^–^N was negative correlated with GY directly (path coefficients = 0.41, and -0.42, respectively). N fertilizer application rate indirectly affected GY via SMBN and NO_3_^–^N contents. SMBN and SMBC did not affect GY directly but correlated with GY through influence on SOC content. These cumulative data showed that the contributions of N fertilizer application to GY were through the regulation of different soil properties in low, moderate, and high fertility soils.

## 4 Discussion

### 4.1 The response of grain yield and nitrogen uptake to nitrogen fertilizer application in soils with different levels of fertility

The response of wheat in grain yield, and N uptake by aboveground biomass was different with maize to nitrogen fertilizer application (**Supplementary Figure A1–A2**). In low fertility soil, the grain yield of wheat increased significantly with the increase of N fertilizer application rates, while those of maize significantly increased at first, but decreased significantly when N application rate exceeded 150 kg N hm^-2^. The main reason was that wheat (a C3 crop) had a relatively moderate demand for N while maize (a C4 crop) had an urgent and high demand for N, but excessive N fertilizer could inhibit root vitality and growth of maize, leading to reduced N transfer in the later stage (Cai, 2010; Zhuang, 2016). In moderate and high fertility soil, the grain yield (2.4%–14.6%), and N uptake by aboveground biomass (15.2%–31.7%) of wheat showed a lower increase compared to maize (188.9%–214.2%, and 45.3%–134.7%, respectively), resulting in a significant decrease of N recovery rate with the increase of N fertilizer application (**Supplementary Figure A3**). This indicated that N fertilizer application had no significant effect on wheat yield, possibly because the precipitation was low in wheat season, which was not conducive to N transport for wheat uptake. Therefore, our subsequent focus primarily centered on determining the appropriate N application rate of maize for soils with varying fertility levels, combining the relationship between grain yield, N recovery efficiency, and N fertilizer application rates. And we investigated the key mechanisms that N fertilizer affects grain yield from the perspective of soil properties.

In this study, the highest grain yield was achieved with 75 kg N hm^-2^ treatment in high fertility soil and 150 kg N hm^-2^ in moderate fertility soil (**Figure 1**), indicating that the amount of N fertilizer applied should be reduced after improving soil fertility from moderate to high. The absence of a significant difference in crop yield among the N75–N187 treatments in high fertility soil served as further confirmation that excessive application of N fertilizer can lead to N loss risk to the environment. In this case, it could be concluded that improvement of soil fertility is an important management practice for the implementation of N fertilizer reduction or zero growth, beyond the “4R” stewardship (“right fertilizer type, “right fertilization amount”, “right fertilization time”, and “right fertilization place”) (Cui et al., 2013; Liu et al., 2016b). In China, approximately 46.8% (63 million hectares) of cropland is classified as moderate fertility soil (Bulletin of the Ministry of Agriculture and Rural Affairs, 2020b), with an average N fertilization application amount of 185 kg hm^-2^ per season (Cui et al., 2018). By upgrading half of the moderate fertility soil to high fertility soil and optimizing the maximum N recovery efficiency (NRE) through a reduction of 35 kg N per hectare compared to current levels of N fertilizer application (185 kg hm^-2^), it would be possible to achieve a savings amount of 1.10 Mt of N fertilizer.

The grain yield in low fertility soil was less than 2 t hm^-2^, and was not improved even after increasing the application rate beyond 112 kg N hm^-2^ (**Figure 1**), suggesting that there is a limitation imposed by the soil itself, which restricted crop growth and inhibited the absorption of N even when sufficient N fertilizer was supplied. In this study, the pH of low fertility soil was 5.35 (**Table 1**), which could potentially limit nutrient transformation and plant growth. Furthermore, SOC mineralization and accumulation content were lower in low fertility soil than in moderate and high fertility soils, which further restricted nutrient transformation (Wang et al., 2019c). Nutrient depletion and soil fertility reduction are the primary causes of low productivity across 33% of global land area (Hüttl and Frielinghaus, 1994; Bini, 2009). For instance, 30 million hectares of the China’s arable land are classified as low fertility soil, of which acidified, salinized, and barren soils accounted for 14 million, 10 million, and 6 million hectares, respectively (Bulletin of the Ministry of Agriculture and Rural Affairs, 2020b). Therefore, conducting thorough investigations of low fertility soil and implementing targeted improvements based on identified obstacles is a fundamental strategy for enhancing the productivity of these soils.

In this study, the N uptake by crop was a little bit higher than the N fertilizer applied in high fertility soil (**Figure 2**). We supposed that the main reason was that the N fertilizer application rate (187.5 kg hm^-2^) was lower than that (225 kg hm^-2^) of conventional fertilization (Cai et al., 2021). Moreover, N in high fertility soil could be rapidly transformed between different forms, which may better meet the growth of crops, and thus promote the absorption of N (Li et al., 2021; Tao et al., 2018).

We also found that, compared to low fertility soil, high fertility soil was primarily conducive to grain production rather than stover formation (**Figure 1**). Interestingly, the harvest index in low fertility soil was 0.12–0.19, while it ranged from 0.27 to 0.33 in high fertility soil (data not shown). The harvest index of high fertility soil was basically consistent with that (0.29–0.38) of previous studies (Li, 2009 and Yan et al., 2013), while those in low fertility soil are lower than those in previous results, which could be attributed to a lower content of soil organic carbon (7.67 g kg^-1^) compared to what was mentioned in previous studies (9.39 g kg^-1^). Therefore, compared to low fertility soil, high fertility soil primarily benefited grain production rather than stover formation. Firstly, we supposed that nutrient storage in high fertility soil could meet the demand of crops in the late reproductive growth stage. Secondly, high fertility soil could promote grain yield by comprehensively increasing both the number of kernels per ear and thousand kernel weight because of the continuous N mineralization, which reduced N stress and the sink strength of grain (Liu et al., 2016a; Ning et al., 2018). Finally, high fertility soil could increase the distribution of dry matter in the grains because of a high grouting rate for a greater length of time and the high transportation and accumulation efficiency of post-anthesis assimilates (Zhang et al., 2021). Therefore, the partitioning of biomass between grain and stover is one of the mechanisms that influences grain production, and requires greater attention in future studies.

### 4.2 Appropriate nitrogen fertilizer application rate for different levels of soil fertility

The grain yield (GY), corresponding N fertilizer application rate, and N recovery efficiency was illustrated in three scenarios of maximum GY, 95% of maximum grain yield, and maximum N recovery efficiency (**Table 2**). For moderate fertility soil, the applicable N fertilizer rate was 140 kg hm^-2^, which resulted in both 95% of maximum grain yield and the highest N recovery efficiency. For rates higher than 140 kg N hm^-2^, additional 40 kg N hm^-2^ (cost $26.8) would be needed to achieve 240 kg hm^-2^ gain yield (benefit $36.0). These findings suggested that the primary objective in moderate fertility soil should be determining the most appropriate N fertilizer application rate rather than endeavor to achieve maximizing yield, in order to balance the yield benefit and environmental cost. For fields with high soil fertility, both the highest grain yield and N recovery efficiency were observed at 131 kg hm^-2^. This was higher than the relatively high yield in **Figure 1** (75 kg N hm^-2^). However, N fertilizer application higher than 75 kg N hm^-2^ improved the N accumulation in the crop (**Figure 2**). We supposed it would benefit soil N conservation and grain quality, such as starch with high viscosity and low retrogradation tendency (Wang et al., 2019a).

Our results indicated that an appropriate N fertilizer application rate is important for sustainable grain production since crop yield and N recovery efficiency increases become negligible and may even begin to decrease when above this rate (**Figure 4**). The appropriate N fertilizer application of this study (ranged from 97.5 kg hm^-2^ to 140 kg hm^-2^) were slightly lower than those (170 kg hm^-2^ to 235 kg hm^-2^) of previous studies (Ren et al., 2022; Zou et al., 2022). This could be attributed to the discrepant climate condition, soil fertility, and cropping systems. In this study, the growing season of maize spans from May to August, and is characterized by high levels of precipitation and temperature, resulting in increased soil moisture, facilitating the mass flow and diffusion of N towards the plant root, thus enhancing N recovery efficiency and reduction of the N fertilizer application rate (Lobell and Field, 2007). Furthermore, the red soil area has lower organic matter and nutrient content, and a finer texture than the average level nationwide (Alotaibi et al., 2018; Zhang et al., 2020), which could lead to a lower N uptake capacity.

### 4.3 Factors influencing maize grain yield in soils with different levels of fertility

Our study suggested that grain production responded via distinct pathways to N fertilization in for the different levels of soil fertility (**Figure 6**). Linear relationship analysis found that grain yield was significantly correlated with both SMBC and SMBN for all soils, while with SOC content or pH value in a specific fertility soil (**Figure 5**). Results from structural equation modeling further indicated that N fertilizer application rate directly correlated with SOC content, which might affect grain production via soil microbial biomass in low fertility soil. It was reported that microorganisms were highly N use efficient at low N concentrations (Kuzyakov and Xu, 2013), and the higher N availability with fertilizer input increased microbial efficiency and N retention in soil (Wang et al., 2023; Wu et al., 2023). In addition, increasing microbial biomass increased grain yield by mineralizing and providing a large amount of available nutrients for crop growth (Ma et al., 2016). Therefore, microbial biomass was important in increasing crop yield, manure or straw should be applied to low fertility soils to effectively stimulate microbial activity and increase the accumulation of microbial biomass, to further improve soil fertility and crop yield.

Grain yield was significantly and positively correlated with SMBC, and N fertilizer application rate significantly correlated with SMBC content both directly and via pH value in moderate fertility soil. It should be noted that there was significant relationship between soil pH, SMBN and nitrate content, indicating that N transformation played an important role. Previous research has shown that excessive application of N fertilizer to red soil resulted in acidification, increasing the accumulation of available N and phenolic acids, but limiting crop growth (Cai et al., 2015; Zhang et al., 2022). In light of this, the application of manure has been found to be an effective strategy for mitigating the adverse effects of soil acidification (Cai et al., 2015). As such, the incorporation of manure should be considered for moderate fertility soils, with particular attention being paid to the interplay between N fertilizer and soil acidification.

In high fertility soil, grain yield responded to N fertilizer directly via NO_3_^–^N content and indirectly via SOC (**Figure 6C**). Grain yield was negatively related to soil residual NO_3_^–^N, which could be attributed to the fact that the increased crop biomass in high-fertility soil resulted in greater uptake and removal of NO_3_^–^N, leading to lower levels of residual NO_3_^–^N (Liu et al., 2017). It is important to note that soil nutrients accounted for only 50% of the grain yield variation, while management strategies, such as varieties and cultivation technique, play an important role in plant growth in soils with no nutrient limitation. For instance, Ren et al. (2023) found that the yield of superior cultivated varieties could be increased by 4% in high fertility soil with high N fertilizer application rate. In addition, a high planting density of 10.5 plants m^−2^ combined with narrow inter-row spacing could lead to a significant maize grain yield increase because the lower yield per plant was fully compensated by the higher planting density (Testa et al., 2016). In general, a comprehensive understanding of yield regulation mechanisms for different levels of soil fertility based on soil properties would facilitate the development and implementation of effective management strategies for improving soil fertility, maximizing crop yield and N use efficiency, while minimizing N loss, would be achievable.

## 5 Conclusion

This study examined the response of maize grain yield (GY) and N recovery efficiency (NRE) to N fertilizer in soil with different levels of fertility. The results clearly showed that soil fertility improvement increased maize yield, and it primarily benefited grain production rather than stover formation. The highest NRE was recorded at N150 but decreased at N187, confirming the negative impact of excessive N fertilizer application on crop N uptake for all three levels of soil fertility. The appropriate N fertilizer application rates for low, moderate, and high fertility soils were determined as 97.5–140 kg hm^-2^, based on three scenarios of maximum GY, NRE, and 95% of maximum GY. Structural equation modeling revealed that both SMBC and SMBN strongly correlated with GY, but the pathways by which it influenced yield (such as SOC, or pH, or NO_3_^–^N content) were different for each level of soil fertility. These results can guide the application of N fertilizer to optimize crop yield and N use efficiency when improving soil fertility, with potential implications for food and ecological security.

## Data availability statement

The original contributions presented in the study are included in the article/**Supplementary Material**. Further inquiries can be directed to the corresponding authors.

## Author contributions

HZ: Conceptualization, Formal analysis, Methodology, Writing – original draft, Writing – review & editing. DL: Conceptualization, Methodology, Resources, Writing – review & editing. KR: Formal analysis, Visualization, Writing – review & editing. LL: Formal analysis, Visualization, Writing – review & editing. WZ: Writing – review & editing. YD: Conceptualization, Funding acquisition, Methodology, Supervision, Writing – review & editing. CL: Writing – review & editing.
