# Supplementary material for: Response of maize yield and nitrogen recovery efficiency to nitrogen fertilizer application in field with various soil fertility
Source: Front Plant Sci. 2024 Feb 27;15:1349180. doi: 10.3389/fpls.2024.1349180 (PMC10935998; doi:10.3389/fpls.2024.1349180)
Supplement: Supplementary file 1 [file DataSheet_1.docx]

**Supplement information**

**Table A1. Soil properties of soil with low, moderate and high fertility (Post maize harvest in 2020)**

| Soil fertility | Nitrogen fertilizer treatment | pH | SOC (g kg^-1^) | NH_4_^+^-N (mg kg^-1^) | NO_3_^-^-N (mg kg^-1^) | Mineral N (mg kg^-1^) | SMBC (mg kg^-1^) | SMBN  (mg kg^-1^) |
| --- | --- | --- | --- | --- | --- | --- | --- | --- |
| Low | N0 | 4.83±0.02a | 5.44±0.09d | 2.46±0.21a | 1.76±0.25bc | 4.23±0.2bc | 57.23±2.36c | 6.11±0.43b |
|  | N75 | 4.78±0.05a | 5.39±0.05d | 2.3±0.11a | 1.24±0.17c | 3.54±0.07c | 61.93±1.7bc | 7.87±0.24b |
|  | N112 | 4.98±0.06a | 7.2±0.05a | 2.56±0.24a | 1.4±0.05c | 3.96±0.26c | 87.02±2.81a | 10.66±0.74a |
|  | N150 | 4.85±0.13a | 5.97±0.07c | 2.91±0.4a | 2.23±0.14b | 5.14±0.36ab | 66.21±1.46b | 12.34±0.66a |
|  | N187 | 4.87±0.16a | 6.47±0.05b | 2.33±0.1a | 3.49±0.37a | 5.82±0.35a | 57.99±1.36c | 11.9±0.53a |
| Moderate | N0 | 6.74±0.1c | 8.03±0.07ab | 1.93±0.31a | 2.41±0.2b | 4.34±0.36b | 62.09±1.41c | 10.94±1.08b |
|  | N75 | 7.22±0.07ab | 8.13±0.09a | 1.76±0.09ab | 3.28±0.1b | 5.04±0.19ab | 99.63±2.41b | 14.89±0.55a |
|  | N112 | 7.44±0.13a | 8.01±0.09ab | 0.96±0.17b | 4.62±0.45a | 5.57±0.31a | 101.57±3.16b | 18.12±0.53a |
|  | N150 | 7.10±0.05b | 7.68±0.1b | 1.53±0.23ab | 3.65±0.33ab | 5.17±0.27ab | 97.48±2.14b | 16.36±0.75a |
|  | N187 | 7.23±0.03ab | 7.91±0.05ab | 1.75±0.1ab | 3.42±0.37ab | 5.17±0.29ab | 109.77±0.1a | 15.67±0.91a |
| High | N0 | 6.35±0.08a | 15.91±0.07ab | 1.96±0.05a | 4.28±0.28b | 6.24±0.24b | 194.43±3.58a | 26.41±0.33a |
|  | N75 | 6.27±0.07a | 16.13±0.14a | 2.07±0.05a | 4.25±0.29b | 6.31±0.28b | 194.59±3.91a | 25.1±1.02a |
|  | N112 | 6.23±0.06a | 15.8±0.09ab | 1.84±0.19a | 3.24±0.22c | 5.08±0.1c | 188.97±3.62a | 20.36±1.08b |
|  | N150 | 6.11±0.04a | 15.85±0.05ab | 1.8±0.13a | 5.02±0.19ab | 6.82±0.33ab | 197.71±6.70a | 25.06±0.66a |
|  | N187 | 5.71±0.07b | 15.56±0.07b | 2.06±0.12a | 5.25±0.02a | 7.31±0.13a | 134.51±3.35b | 15.25±0.63c |

Notes: values are the mean ± standard deviation (n = 3). Low, Moderate, and High represent low fertility soil, moderate fertility soil, and high fertility soil, respectively. Different lowercase letters indicate significant difference at 5% level for the same fertility soil.

**Figure captions**

**FIGURE A1.** Grain yield of wheat (a) and aboveground biomass of wheat (b) for three levels of soil fertility with N fertilizer application gradients.

**FIGURE A2.** Nitrogen uptake by wheat aboveground biomass for three levels of soil fertility with N fertilizer application gradients.

**FIGURE A3.** Nitrogen recovery efficiency of wheat for three levels of fertility soils with N fertilizer application gradients.


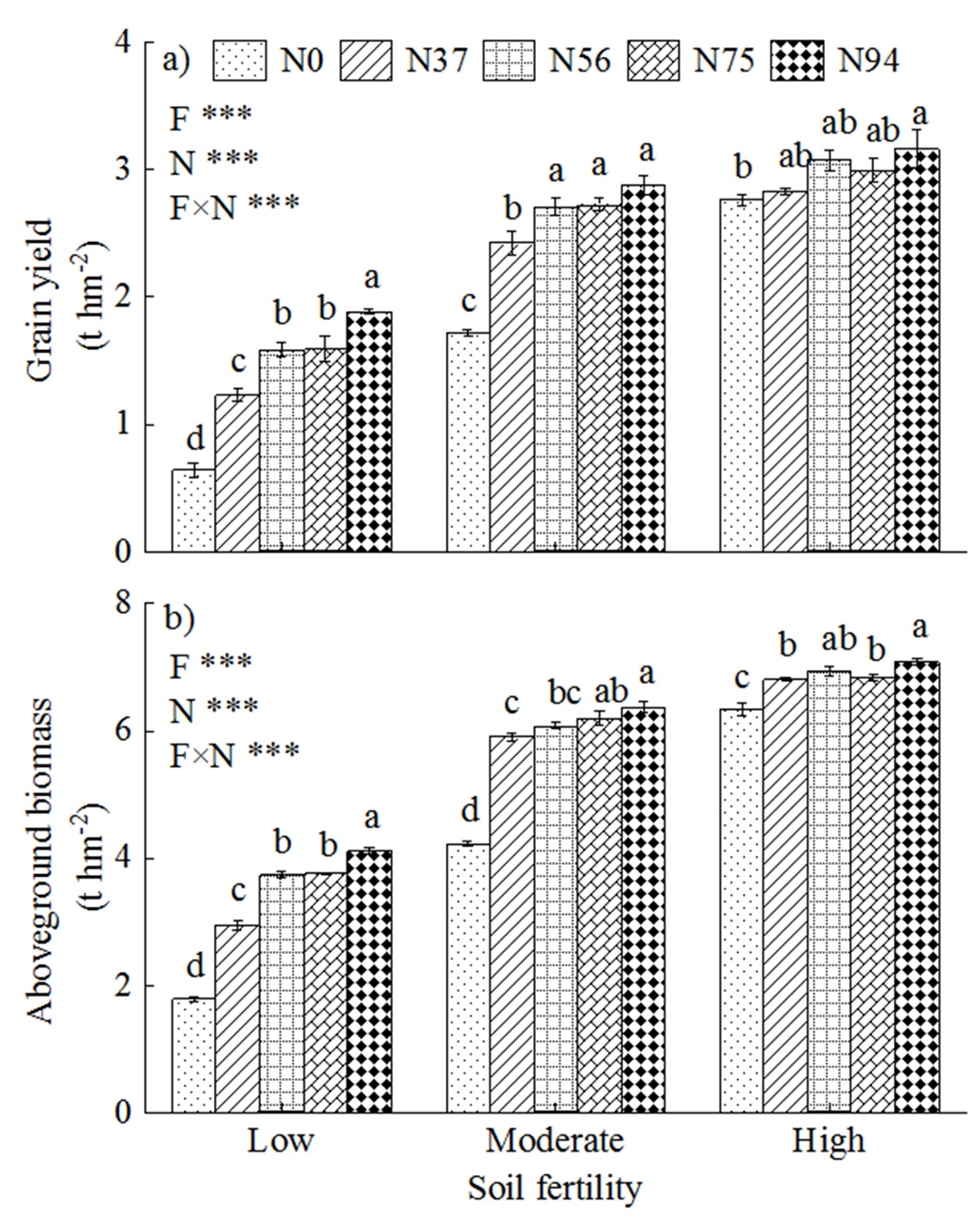


**FIGURE A1.** Grain yield of wheat (a) and aboveground biomass of wheat (b) for three levels of soil fertility with N fertilizer application gradients

Notes: N0, N37, N56, N75, and N94 indicate nitrogen fertilization rates of 0, 37.5, 56.25, 75, and 93.75 kg N hm^-2^, respectively. Low, Moderate, and High represent low fertility soil, moderate fertility soil, and high fertility soil, respectively. Different lowercase letters indicate significant difference at 5% level in the same fertility soil. F, N and F×N represent soil fertility, nitrogen fertilizer application and interaction effect of soil fertility and nitrogen fertilizer, respectively. *** represent *P* < 0.001.


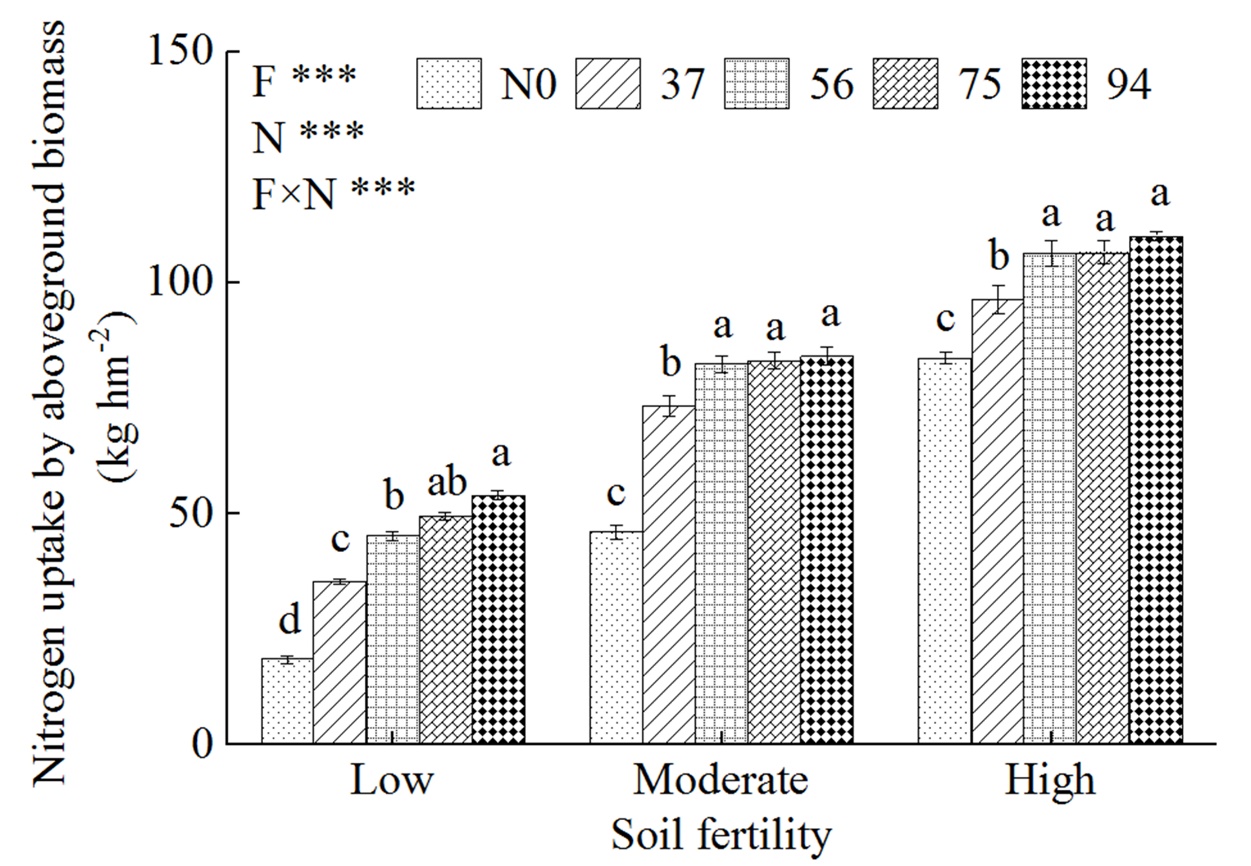


**FIGURE A2.** Nitrogen uptake by wheat aboveground biomass for three levels of soil fertility with N fertilizer application gradients

Notes: N0, N37, N56, N75, and N94 indicate nitrogen fertilization rates of 0, 37.5, 56.25, 75, and 93.75 kg N hm^-2^, respectively. Low, Moderate, and High represent low fertility soil, moderate fertility soil, and high fertility soil, respectively. Different lowercase letters indicate significant difference at 5% level in the same fertility soil. F, N and F×N represent soil fertility, nitrogen fertilizer application and interaction effect of soil fertility and nitrogen fertilizer, respectively. *** represent *P* < 0.001.


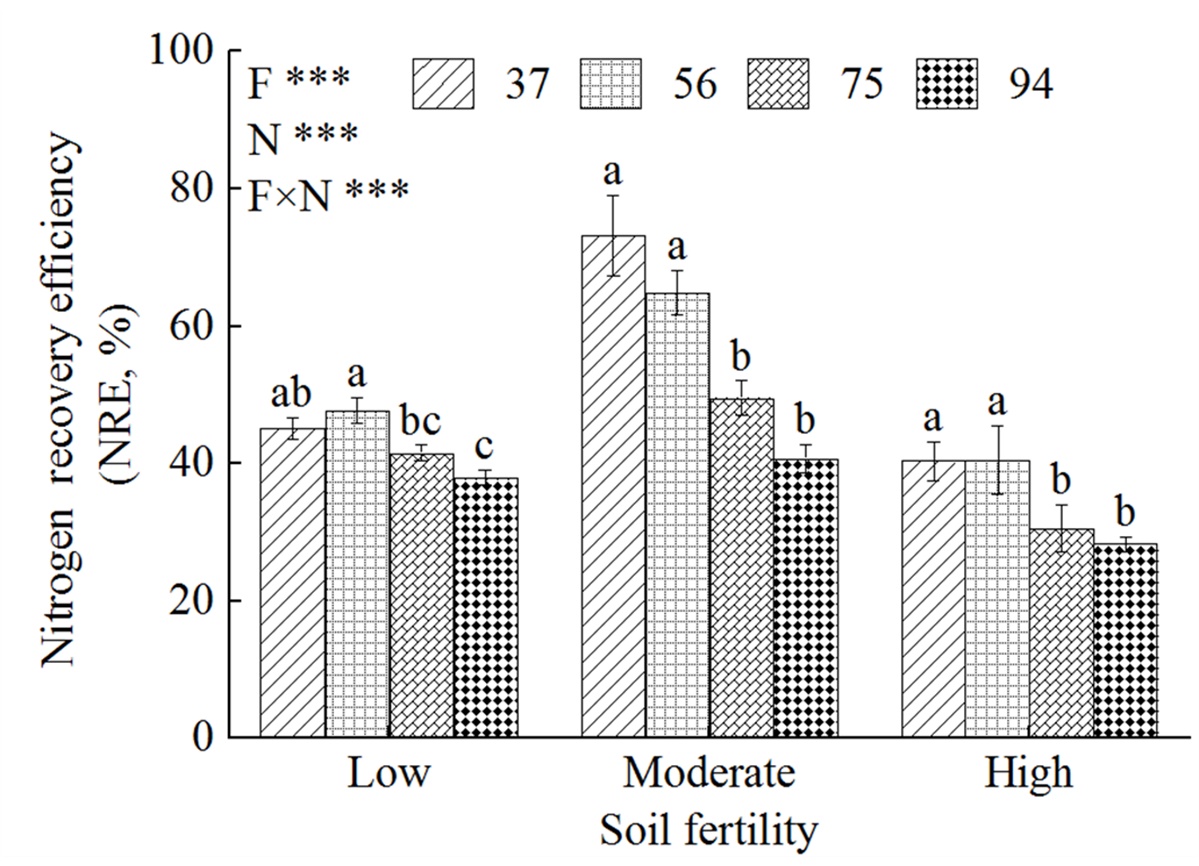


**FIGURE A3.** Nitrogen recovery efficiency of wheat for three levels of fertility soils with N fertilizer application gradients.

Notes: N0, N37, N56, N75, and N94 indicate nitrogen fertilization rates of 0, 37.5, 56.25, 75, and 93.75 kg N hm^-2^, respectively. Low, Moderate, and High represent low fertility soil, moderate fertility soil, and high fertility soil, respectively. Different lowercase letters indicate significant difference at 5% level in the same fertility soil. F, N and F×N represent soil fertility, nitrogen fertilizer application and interaction effect of soil fertility and nitrogen fertilizer, respectively. *** represent P < 0.001.
